# Role of blood glucose and fat profile in lung function pattern of Indian type 2 diabetic subjects

**DOI:** 10.1186/s40248-019-0184-5

**Published:** 2019-07-01

**Authors:** Morteza A. Khafaie, Sundeep S. Salvi, Chittaranjan S. Yajnik, Fakher Rahim, Behzad Khafaei

**Affiliations:** 1 Social Determinants of Health Research Center, Ahvaz Jundishapur University of Medical Sciences, Ahvaz, Iran; 2 Department of Public Health, Faculty of Health, Ahvaz Jundishapur University of Medical Sciences, Ahvaz, Iran; 3 Chest Research Foundation (CRF), Pune, Maharashtra India; 4 King Edward Memorial Hospital Research Center, Pune, Maharashtra India; 5 Thalassemia and Hemoglobinopathy Research Centre, Health Research Institute, Ahvaz Jundishapur University of Medical Sciences, Ahvaz, Iran; 6 Department of Statistics, Omidieh Branch, Islamic Azad University, Omidieh, Iran

**Keywords:** Respiratory distress syndrome, Diabetes mellitus, Hyperglycaemia, Fat profile; risk factors

## Abstract

**Background and objectives:**

It has been hypothesized that changes in lung function can occur in patients with diabetes. Nevertheless, it is unclear how much of this correlation links with biomarkers of metabolism disorder. We have investigated the association between hypoglycaemic and fat profile with lung function in Indian diabetic subjects.

**Design:**

Prospective observational study.

**Setting:**

Diabetes care unit of King Edward Memorial (KEM) hospital.

**Patients:**

Out of 465 patients who agreed to participate in this study, valid lung function data were available from 347 Type 2 diabetic subjects.

**Measurements:**

Pulmonary function test including predicted forced vital capacity (% FVC), predicted forced expiratory volume in 1 second (% FEV_1_) and FEV_1_/FVC ratio were assessed. We also examined fat profile, glucose, HbA1c, hemoglobin and other hematological parameters.

**Results:**

Four hundred sixty-five subjects aged 55 ± 11 participated in the study. Predicted forced vital capacity, % FEV_1_ and FEV_1_/FVC ratio was 85.88 ± 13.53, 85.87 ± 14.06 and 82.03 ± 6.83, respectively. Also, approximately 8 to 17% of the participant reported having at least one chronic respiratory symptom or lung disease. We found that high glycaemic measures (i.e. fasting and post-meal plasma glucose) are linked with dyspnea. In addition, HDL (high-density lipoprotein) concentration was directly associated with % FVC.

**Conclusions:**

It is difficult to draw a clear conclusion about the cause-effect relationship or clinical impact based on this study alone. However, identification of clinically meaningful elements for developing a screening program is critical.

## Background

Diabetes mellitus (DM) describes a metabolic disorder of multiple etiologies characterized by chronic hyperglycaemia with disturbances of fat, protein and carbohydrate metabolism due to impairment in insulin secretion, insulin action, or both [1]. In this condition, the body needs to increase its medication and eventually insulin infusion is necessary [2]. In many developing regions such as India, thus, lack of appropriate medical care especially due to health gradient leads these populations to develop diabetes at lower ages and BMI [3]. Earlier onset of diabetes may result in the development of severe complications [4].

The lung is one of the target organs in diabetes of which the damage is quite subclinical and often ignored by patients and physicians [5]. Respiratory distress is a public life-threatening condition with an estimated 150,000 cases in the United States annually. Some evidence, but not all, proposed DM as a risk modifier for respiratory distress, especially acute respiratory distress syndrome (ARDS). Some studies have reported DM to be associated with lower development of respiratory distress [6–8], whereas others found that DM increases the risk of this disorder [9].

A high proportion of patients with chronic respiratory disease have simultaneous metabolic disorders [10–12]. Although some evidence has been suggested, chronic respiratory disorders (i.e. lung volume, pulmonary diffusing capacity, control of ventilation, bronchomotor tone, and neuroadrenergic bronchial innervation) are a risk factor for diabetes [13, 14], others reported diabetes as a risk factor for respiratory disorders [15]. Furthermore, metabolic disorders, especially diabetes that generally manifested with obesity, are associated with a substantial loss of pulmonary function in a restrictive pattern [16–19].

Many hypotheses have emerged to elucidate the pathogenesis of the diabetic lung injury, and characteristic “diabetic lung” [5]. Oxidative stress, non-enzymatic glycation of proteins, and the polyol pathway have been recognized to be elaborated in the etiology of diabetic lung injury [20]. We speculate that glycaemic control and fat profile that linked with collagen and elastin changes [21] can lead to significant structural changes in the respiratory system.

### Patients and methods

#### Study design and participation

Type 2 diabetic subjects attended diabetes care unit of King Edward Memorial (KEM) hospital, between March and December 2011, and consented to participate in the study (*N* = 465), and were administered a standard questionnaire (available online as supplementary material [22]). The questionnaire was designed to capture demographic data, medical history of the patient and data related to the duration of activity, the source of indoor air pollution, chronic respiratory symptoms (CRS), asthma and COPD. CRS included: chronic cough (cough or phlegm apart from common cold that has been accruing for at least 3 months of the year for the last 2 years), dyspnea (any attack of shortness of breath with wheeze, apart from common colds in the last 12 months), wheezing (a wheeze for at least 6 months of the year, apart from common colds), chest tightness (feeling of tightness in the last 12 months in their chest), and allergy (symptoms such as hay fever or any other condition making the nose runny or stuffy, apart from common colds, associated with redness of eyes, itching, burning, and eczema present in most days of the week).

Blood samples also were drawn to measure various biochemical parameters, including fat profile, glucose, HbA1c, hemoglobin and other hematological parameters. Complete details of the survey design and examination procedures have been published elsewhere [23]. Subjects with history of recent eye surgery, recent abdominal surgery, stroke that is affecting the face, recent myocardial infarction, smoking cigarettes, using short-acting bronchodilator such salbutamol (last 4–6 h) or long-acting agents, having a heavy meal, having flu-like illness or cold could affect the spirometry results were excluded from the study.

### Procedure

Subjects were asked to remove tight clothing such as ties and belts and perform the spirometry test three times (acceptable and repeatable [22]), using ultrasonic spirometer (ndd, Switzerland), and the best ones of the three results were taken into consideration. The examinations were performed with the subject in a sitting position wearing a nose clip and using a disposable mouthpiece. The quality of spirometry measurements was controlled by chest research foundation (CRF) specialists. Referees reviewed all loop recordings and excluded those without at least three satisfactory tests (see details in [24]. We interpreted the subject’s lung function as 90% of the European Community for Steel and Coal (ECSC) value as a reference [25].

### Statistical analyses

For all models, the dependent variables were percent predicted FVC (%FVC), percent predicted FEV_1_ (% FEV_1_), cough, dyspnea, allergy, history of asthma or COPD. Also, each biomarker (i.e. FPG, 2_hrs_PG, Hemoglobin, HbA1c, total cholesterol, LDL cholesterol, HDL cholesterol, triglyceride, and WBC), a series of regression models were performed independently. Initial models assessed the linear association of the biomarkers with PFT (i.e. %FVC and %FEV_1_) adjusting for potential confounding factors including age, gender, BMI, cigarette smoking and medication (i.e. aspirin, statin, and TZD) as appropriate. Results are presented as % change in mean % predicted PFT for 1 SD μg/m^3^ increments in biomarkers using the following formula: %change = (Coef./mean)1 SD*100, where, Coef. = coefficients of association, Mean = average % predicted the value of FEV_1_ and FVC, 1 SD = 1 Standard Deviation of biomarkers. Further, we studied the odds ratio of having a chronic cough, dyspnea, asthma and COPD for 1 SD increase in biomarkers, using logistic regression. Exponential of estimated coefficients (equal to odd ratio) was reported. The significance threshold of *p* = 0.05 was used in all analyses. All statistical analyses were performed using STATA version 11.1 software (STATA Corporation, College Station, TX).

## Results

Descriptive characteristics of the study population are depicted in Table 1. Out of 465 patients who agreed to participate, valid lung function data were available from 347 diabetic subjects. There were no significant differences between 347 patients for whom lung function was available and the rest of subjects in terms of age, gender, and BMI. More than half of diabetic subjects patients were on at least one of an anti-inflammatory agent such as aspirin, statins, and TZD (Thiazolidinedione).

**Table 1:** Descriptive characteristics of study population

Characteristics	*N*	Diabetic subjects
Male	465	268 (58)
Age, yrs.*	465	54.58 (11.11)
BMI, kg/m^2^*	462	26.71 (4.08)
Waist-Hip ratio*	314	0.96 (0.10)
FVC, Liters*	374	2.49 (0.52)
FEV_1_, Liters*	374	2.02 (0.44)
% FEV_1_/FVC*	374	82.03 (6.83)
% predicted FVC	374	85.88 (13.53)
% predicted FEV_1_	374	85.87 (14.06)
Smoking	465	34 (7.31)
Tobacco	465	97 (21.00)
Alcohol	462	76 (16.45)
Non-vegetarian*	465	295 (63.00)
Chronic cough*	465	51 (11.00)
Dyspnea_(MRC1–5)_*	465	145 (31.00)
Wheezing*	465	40 (8.60)
Tightness*	465	39 (8.40)
Allergy symptom	465	81 (17.40)
COPD history	465	
self*		10 (2.40)
family		6 (1.50)
Asthma history	465	
self*		17 (4.20)
family *		66 (16.20)
TZD	465	38 (11.50)
Aspirin	465	162 (48.00)
Statin	465	190 (56.5)

Data is shown as n (%) and mean (SD); The difference between groups was tested by t-test and Chi-square, as appropriate; The test was adjusted for age, gender, and BMI as appropriate

*indicate *p* < 0.05

### Blood biomarkers

Data obtained from patients recorded profiles up to 1 month before enrollment for the lung function test. All variables were normally distributed except triglyceride and WBC. Therefore, these variables were logarithmically transformed (Table 2).

**Table 2:** Biomedical indicator of systemic inflammation, glycaemic control and fat profile of diabetic subjects

	Women		Men		Total	
	N	Mean (SD)	N	Mean (SD)	N	Mean (SD)
FPG, mg/dl	189	146.88 (54.66)	252	141.37 (52.14)	441	441; 143.73 (53.25)
2_hrs_PG, mg/dl	189	215.85 (68.34)	254	218.61 (73.52)	443	443; 217.43 (71.29)
Hemoglobin, g/dl*	122	12.37 (1.52)	155	14.06 (2.14)	277	277; 13.32 (2.07)
HbA1c, %	112	8.86 (2.21)	157	8.84 (2.15)	269	269; 8.85 (2.17)
Cholesterol	122	157.30 (41.92)	149	151.16 (40.54)	271	271; 153.93 (41.21)
Triglyceride	141	133.47 (60.10)	166	135.59 (92.28)	307	307; 134.61 (79.03)
HDL*	121	41.30 (8.51)	154	36.47 (8.57)	275	275; 38.60 (8.86)
WBC × 10^^9^/Liter*	93	8.82 (2.55)	120	7.74 (1.72)	213	213; 8.21 (2.18)

The differences between groups were tested using t-test and Wilcox on Man-Whitney as appropriate

*FPG* fasting plasma glucose, *2*_*hrs*_*PG* 2 h post meal plasma glucose, *HDL* High-density lipoprotein

*Indicate *p* < 0.05

Women had lower Hb (12.37 for women vs. 14.06 for men, *p* < 0.05), and higher WBC, and triglyceride concentration. All the markers were inversely related to age (younger patients had a higher value). In addition, we found BMI inversely related to HDL concentration and expectedly patient on statin treatment had lower cholesterol concentration compared to those not on statin treatment. The inverse association between aspirin and cholesterol is due to the fact that in this clinic the statins proportion combined with aspirin.

### Association between blood biomarkers and chronic respiratory symptoms (CRS)

The association between blood biomarkers and CRS is shown in Table 3. We have observed that only a weak association between biomarkers of glycaemic measure (i.e. FPG and 2_hrs_PG) and dyspnea exist. For instance, 10 mg/dl increase in FPG was associated with a 7% increase in the risk of dyspnea. There were no significant associations with other CRS component.

**Table 3:** Association of selected biomarkers of glycaemic control, fat profile, and systemic inflammation with chronic respiratory symptoms (CRS)

	Cough *N* = 62 (7.17%)	Dyspnea *N* = 197 (22.77%)	Allergy sym. *N* = 112 (12.95)	Asthma/COPD *N* = 26 (3.01%)
FPG^a^	1.01 (1.00–1.02)	**1.01 (1.00–1.02)**	1.00 (1.00–1.01)	1.00 (0.98–1.01)
2_h_PPG^a^	1.00 (1.00–1.01)	**1.00 (1.00–1.01)**	1.00 (0.99–1.00)	1.00 (0.99–1.01)
Hemoglobin^b^	1.05 (0.81–1.35)	0.96 (0.77–1.20)	0.92 (0.71–1.18)	1.03 (0.70–1.50)
HbA1c^a^	1.15 (0.94–1.41)	1.02 (0.87–1.19)	0.99 (0.81–1.19)	0.91 (0.69–1.20)
Cholesterol^c^	1.00 (0.99–1.01)	0.99 (0.98–1.00)	1.00 (0.99–1.02)	1.01 (0.99–1.02)
Triglyceride^a^	1.70 (0.76–3.83)	1.19 (0.65–2.17)	0.51 (0.25–1.17)	1.34 (0.47–3.84)
HDL^d^	0.98 (0.92–0.92)	0.98 (0.94–1.02)	1.04 (0.99–1.08)	1.04 (0.98–1.12)
WBC^a^	3.09 (0.51–18.92)	1.37 (0.37–5.12)	0.35 (0.06–1.96)	1.37 (0.12–16.10)

*FPG* Fasting plasma glucose, *2*_*hrs*_*PPG* 2 hrs. Post meal plasma glucose

All variables are Odds Ratio (95% CI)

^a^Adjusted for age, BMI, and smoking

^b^Adjusted for age, gender BMI, and smoking

^c^Adjusted for age, BMI, smoking, aspirin, and statin

^d^Adjusted for age, gender, BMI, smoking, and aspirin

Bold “OR” are significant at *p* < 0.05

### Association between blood biomarkers and lung function

No significant relationship between blood biomarkers and measures of lung function were documented except between HDL and %FVC (but not with FEV_1_, see Table 4). In addition, one SD (=8.86 mg/dl) increase in HDL was associated with a 6.22% (0.18–12.27) increase in %FVC.

**Table 4:** Association between lung function (measured and % predicted) and selected blood biomarkers

	% FEV_1_	%FVC
FPG, *n* = 329	− 0.01 (− 0.05–0.03)	0.00 (− 0.04–0.03)
2_hrs_PPG, *n* = 334	0.00 (− 0.20–0.03)	0.01 (− 0.01–0.03)
Hemoglobin, *n* = 202	− 0.55 (− 2.06–0.97)	−0.06 (− 1.50–1.38)
HbA1c, *n* = 200	−0.01 (− 1.09–1.06)	−0.22 (− 1.26–0.82)
Cholesterols, *n* = 197	0.02 (− 0.04–0.08)	0.04 (− 0.02–0.10)
Triglyceride^a^, *n* = 234	0.58 (− 4.51–3.35)	−0.98 (− 4.72–2.77)
HDL, *n* = 202	0.00 (− 0.28–0.28)	**0.27 (0.08–0.53)**
WBC, *n* = 160	−1.04 (− 11.59–9.50)	1.60 (− 8.44–11.64)

All variables are Coefficient (95% Confidence Interval)

^a^variables are natural logarithm

Bold “value” are significant at *p* < 0.05

**Fig. 1:**
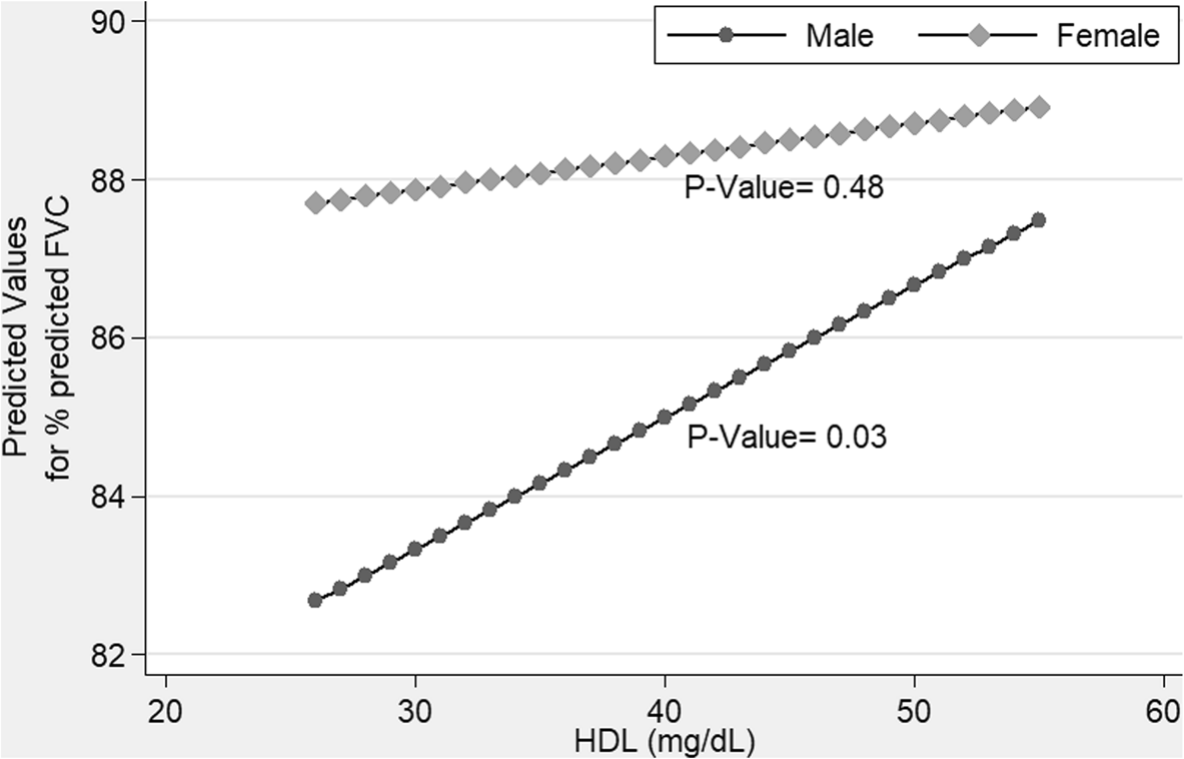
Association between HDL and Forced Vital Capacity among men and women

We did not find any differences in this relationship with age (cut point 46 years), gender, and BMI (either cut point 23 and 25) groups (Fig. 1).

## Discussion

We found that glycaemic measures (i.e. high fasting and post-meal plasma glucose) linked to risk of dyspnea. HDL concentration was directly associated with %FVC. Unlike a study in Korea [26], we did not find significant age or gender differences in the association between HDL and %FVC (Fig. 1). This association was also independent of overall or central obesity. Our result was robust to additional adjustment for exogenous confounding factors (such as temperature and cigarettes smoke) and consistent with a study from France [27] showing HDL as an independent predictor of %FVC. Also similar to a study from Italy [28], we demonstrate that HDL is associated with impaired lung function in a mainly restrictive pattern. Other studies have also shown an association with metabolic syndrome [16] and low HDL [27] and restrictive, but not an obstructive respiratory pattern.

Mechanisms underlying diabetes lung defect are not fully clear. Glycosylation of protein in a patient with poor metabolic control leads to the accumulation of collagen in lung connective tissue [5]. The lung collagen accumulation and increased stiffness of lung parenchyma and chest wall may cause the restrictive functional defect appearing in lung disease. Studies have suggested that impaired pulmonary function may be a potential risk factor for type 2 diabetes [29], where the underlying mechanisms closely linked to an excess of oxidative stress and chronic low-grade inflammation [30, 31]. Inflammatory markers such as C-reactive protein (CRP) have been associated with impaired pulmonary function among subjects with diabetes [32]. In contrast, HDL may have a positive effect through its role in immune regulation. HDL has been shown to bind to bacterial endotoxin as well as to relieve inflammation [33, 34], suggesting a potential role for HDL in preventing lung tissue damage. In the present study WBC, a marker of nonspecific systemic inflammation was not associated with HDL.

## Conclusion

It is difficult to draw a clear conclusion about the cause-effect relationship or clinical impact based on this study alone. Further studies are required to clarify the causal relationship and to assess clinical outcomes including long-term changes in lung function. Specifically, the finding, if there is heterogeneity in the association between a biomarker of interest and lung function pattern across patients with Asthma and COPD, could be indicative and would provide us with a better understanding of the mechanism of diseases. Since the high rate of respiratory disease is under-diagnosed, identification of clinically meaningful elements for developing a screening program is critical.

## Abbreviations

ARDS: Acute respiratory distress syndrome
CRF: Chest Research Foundation
CRP: C-reactive protein
CRS: Chronic respiratory symptoms
DM: Diabetes mellitus
ECSC: European Community for Steel and Coal
KEM: King Edward Memorial
